# Subtype- and antigenic site-specific differences in biophysical influences on evolution of influenza virus hemagglutinin

**DOI:** 10.1186/1743-422X-9-91

**Published:** 2012-05-08

**Authors:** Stephen J Stray

**Affiliations:** 1 Department of Microbiology, University of Mississippi Medical Center, 2500 N State St, Jackson, MS, 39216, USA; 2 Base Pair Program, University of Mississippi Medical Center, 2500 N State St, Jackson, MS, 39216, USA; 3 Present address: Sally McDonnell Barksdale Honors College, University of Mississippi, Oxford, MS, 38667, USA

## Abstract

**Background:**

Influenza virus undergoes rapid evolution by both antigenic shift and antigenic drift. Antibodies, particularly those binding near the receptor-binding site of hemagglutinin (HA) or the neuraminidase (NA) active site, are thought to be the primary defense against influenza infection, and mutations in antibody binding sites can reduce or eliminate antibody binding. The binding of antibodies to their cognate antigens is governed by such biophysical properties of the interacting surfaces as shape, non-polar and polar surface area, and charge.

**Methods:**

To understand forces shaping evolution of influenza virus, we have examined HA sequences of human influenza A and B viruses, assigning each amino acid values reflecting total accessible surface area, non-polar and polar surface area, and net charge due to the side chain. Changes in each of these values between neighboring sequences were calculated for each residue and mapped onto the crystal structures.

**Results:**

Areas of HA showing the highest frequency of pairwise changes agreed well with previously identified antigenic sites in H3 and H1 HAs, and allowed us to propose more detailed antigenic maps and novel antigenic sites for H1 and influenza B HA. Changes in biophysical properties differed between HAs of different subtypes, and between different antigenic sites of the same HA. For H1, statistically significant differences in several biophysical quantities compared to residues lying outside antigenic sites were seen for some antigenic sites but not others. Influenza B antigenic sites all show statistically significant differences in biophysical quantities for all antigenic sites, whereas no statistically significant differences in biophysical quantities were seen for any antigenic site is seen for H3. In many cases, residues previously shown to be under positive selection at the genetic level also undergo rapid change in biophysical properties.

**Conclusions:**

The biophysical consequences of amino acid changes introduced by antigenic drift vary from subtype to subtype, and between different antigenic sites. This suggests that the significance of antibody binding in selecting new variants may also be variable for different antigenic sites and influenza subtypes.

## Background

Influenza virus undergoes rapid evolution in nature by both genetic shift, where one (or more) of the eight gene segments is exchanged from one virus into another [1], and genetic drift, whereby mutations accumulate in viral genes [2], presumably due to the relatively error-prone replication of the viral RNA. This presents a significant challenge for vaccine design, as new vaccines must be produced almost every year in order to provide the best match with viruses likely to circulate in the coming influenza season. While other potential targets for vaccination to protect against influenza infection are under investigation [3,4], it is likely that vaccines based on the intact surface proteins of influenza viruses will remain in use for the foreseeable future. The activities of both hemagglutinin (HA) and neuraminidase (NA) are essential to viral function, and antibodies recognizing HA and NA are the primary defense against viral infection [5]. Antibodies binding near the receptor-binding site of HA [6,7] or the substrate binding site of NA [8,9] strongly inhibit viral function, so it is presumed that mutations in these binding sites which reduce or eliminate antibody binding confer a significant evolutionary advantage.

Studies of changes occurring in human influenza isolates and the selection of “escape mutant” variant viruses resistant to neutralizing monoclonal antibodies have allowed the delineation of critical neutralizing antigenic sites in both HA and NA [7]. In many cases, a single amino acid change is sufficient to reduce, often drastically, the neutralizing effect of antibody. Studies of interactions between mutant influenza NA and monoclonal antibodies at the biochemical and structural level have revealed at least two classes of binding phenomena; for some antibody-antigen pairs, the contribution of some amino acids is much more important than others in the epitope, presumably because interactions with these amino acids contribute much more to the antibody binding energy [10,11], while for other antibody-antigen pairs, the contribution of each amino acid in the epitope is approximately similar [12,13], suggesting that considerations such as shape complementarity between the binding site on the antibody and the antigenic site is critical to antibody binding. Biophysical analyses of antigen/antibody pairs consisting of either lysozyme and monoclonal antibody or idiotype/anti-idiotype monoclonal antibody pairs suggest that epitopes that are tightly bound by antibody may often have a hydrophic core surrounded by hydrophilic amino acids, suggesting that both entropy and electrostatics are important in antibody binding (reviewed in [14]). It should be noted the total number of antibody/antigen pairs that have been analyzed at the biophysical level remains small, so any generalization must be made with caution.

As first suggested by Darwin [15], evolution is presumably governed by a complex interplay between positive selection for a novel function, such as a new enzyme specificity or escape from antibody binding, and negative selection against those changes which have a deleterious effect on the protein’s structure or critical functions or interactions. To begin to understand the forces shaping the evolution of influenza virus HA, we have examined HA sequences available in the National Center for Biotechnology Information (NCBI) Influenza Database [16]. We reasoned that, if ongoing selection by neutralizing antibodies is important, those residues targeted by neutralizing antibody will continually change over time. Thus, we have made pairwise comparisons between aligned sequences to look for changes in closely related HAs. We have both quantitated the frequency of change of individual amino acids, and attempted to understand how these changes affect the biophysical properties of individual residues within HA. Our studies indicate that the types of changes observed at different antigenic sites vary between influenza subtypes, and between individual antigenic sites in the same HA. We also demonstrate that many HA residues shown by others to be under positive selection at the genetic level [17,18] also have a propensity to undergo changes in biophysical properties. These data may prove useful in developing algorithms to better predict future changes in influenza antigens to improve influenza vaccine design.

## Methods

### Influenza sequences and sequence alignments

Amino acid sequences for the HA1 domain of HA from human clinical H1N1 (n = 531, 1918–2008, i.e. excluding 2009 “Swine-origin” pandemic isolates), H3N2 (n = 968, 1968–2005), and influenza B (n = 209, 1940 – 2007, alignments performed without separating out Victoria and Yamagata lineages). Due to the fact that many sequences did not contain complete sequence data for the HA2 portion of the molecule, analyses were performed solely for the HA1 portion. Amino acid sequences were obtained and a best fit alignment performed using MUSCLE [19], as implemented in the NCBI Influenza Virus Resource (http://www.ncbi.nlm.nih.gov/genomes/FLU, [16]). Incomplete and duplicate sequences were removed prior to alignment where possible. See Additional file 1 for sequence alignments used in this study.

### Pairwise comparison of aligned sequences

Aligned sequences from NCBI were uploaded into Kalignvu (http://msa.sbc.su.se/cgi-bin/msa.cgi, [20]) to produce a dataset containing complete amino acid sequences which were then uploaded into Excel (Microsoft, Renton WA). The absolute number of pairwise changes at each position was determined and divided by the total number of sequences. This was designated *∆abs*, and represents the frequency of any amino acid change at a given position. Note that, under this approach, a single change from the root sequence which is then perpetuated throughout the rest of the sequences in the alignment will have a low value for *∆abs*, whereas a position where different amino acids can occur in different sequences will have a much higher *∆abs.*

### Parameterization and calculation of change in biophysical properties

Each amino acid in the dataset was then assigned values for *∆ASA*_*tot*_*∆ASA*_*np*_, and *∆ASA*_*pol*_ (Table 1, [21,22]). Each amino acid was also assigned a value for net charge at pH 7.0 (*Q*, Table 1) based on the side chain p*K*_*a*_, with completely ionized acidic and basic residues being assigned values of −1 and +1, respectively. For every residue in HA, pairwise changes in each parameter were calculated by subtracting the assigned value from that at the same position in the sequence immediately above it in the alignment table (i.e. the most closely related sequence). The absolute values of these differences were averaged for the same position in all sequences in the alignment table, then normalized to *∆abs* to generate Normalized Change Index (NCI) values for *∆∆ASA*_*tot*_*∆∆ASA*_*np*_*∆∆ASA*_*pol*_ and *∆Q*. Thus, in cases where no change was observed between the two sequences, the numerical value of the difference was zero, but where a difference occurred, the value represents the average magnitude of the difference every time a change occurs. Because of the normalization to *∆abs*, a frequently occurring conservative change can be readily distinguished from a rarer, non-conservative change. Values for *∆abs, ∆∆ASA*_*tot*_*∆∆ASA*_*np*_*∆∆ASA*_*pol*_ and *∆Q* for each amino acid position in HA were analyzed statistically to determine the median, 75^th^ percentile and 90^th^ percentile values for each dataset using Kaleidagraph (Synergy Software). Rapidly changing residues were defined as those residues in the 75^th^ percentile and above in terms of *∆abs.*

**Table 1:** Values for parameters assigned to each amino acid

**aa**	**A**	**R**	**N**	**D**	**C**	**Q**	**E**	**G**	**H**	**I**	**L**
**∆ASA**_**tot**_^**a**^	54.6	199.6	112.9	99.4	92	122.3	124.5	0	144.9	135.9	143.8
**∆ASA**_**np**_	62.9	85.3	28.5	42.2	40.8	46.7	55.9	25.7	99.5	139.5	144.4
**∆ASA**_**pol**_	28.7	151.3	113.3	87	96.1	110.1	103.6	28.7	74.1	28.7	28.7
**Q**^**b**^	0	1.00	0	−1.00	−0.11	0	−1.00	0	0.05	0	0
**aa**	**K**	**M**	**F**	**P**	S	**T**	**W**	**Y**	**V**	**Deleted/missing**^**c**^	
**∆ASA**_**tot**_	155.6	158	172	90.7	71.7	105.4	222.4	190.2	105.6	300	
**∆ASA**_**np**_	122.4	122.1	172	100.8	44.2	74.8	200.5	154.3	113	258.3	
**∆ASA**_**pol**_	70.3	73	28.7	15.6	64.4	63.4	52.4	71.6	26.2	64.4	
**Q**	1.00	0	0	0	0	0	0	0	0	0	

^a^Values for *ΔASA*_*tot,*_*ΔASA*_*np,*_ and *ΔASA*_*pol*_ as in [21]. Note that values for *ΔASA*_*tot*_ are all based on comparison to the surface area of glycine, which is set to 0.

^b^Calculated at pH 7.0, setting completely ionized acids and bases at −1.00 and 1.00, respectively.

^c^Values for deleted or missing amino acids were chosen arbitrarily.

### Structural analysis, assignment of antigenic sites, and statistical analysis

To allow comparison of changes in biophysical parameters with previously defined antigenic sites and the receptor binding pocket, amino acid residues in the crystal structures of H1, H3, and influenza B HA were color-coded to represent NCI values for biophysical parameters (see individual figure legends for structures used in each case), using Mac PyMol (DeLano Scientific LLC). Rapidly changing residues (*∆abs* ≥ 75^th^ percentile) were color-coded based on whether the NCI value of interest fell below the median, was between the 50^th^ and 75^th^ percentile, between the 75^th^ and 90^th^ percentile, or above the 90^th^ percentile for HA1 residues in terms of NCI values for *∆∆ASA*_*tot*_, *∆∆ASA*_*np*_, *∆∆ASA*_*pol*_ and *∆Q*. See figure legends for further details.

Rapidly changing amino acids on the outer surface of the respective HA1 monomers formed surface patches roughly analogous to the previously described neutralizing antigenic sites of H1, H3, and B HA antigenic sites, and were deemed to belong to these antigenic sites. The properties of these antigenic sites were compared statistically by comparing the value of each parameter for all the residues assigned to a particular antigenic site to a dataset comprising all amino acids from the HA1 portion of the same HA molecule not assigned to antigenic sites (non-antigenic site residues). It is assumed that non-antigenic site residues include both amino acids that cannot be altered without deleterious effects on structure or function and residues subject to genetic drift but where antibody-mediated selection is unlikely to occur. The majority of non-antigenic site residues undergoing rapid change are on solvent-exposed surfaces not likely to be accessible to antibody, such as on the back of the monomer. Statistical comparisons were performed using Kruskal-Wallis ANOVA with Dunn’s post- test (GraphPad Prism).

### Effect of alignment on biophysical parameters

To test for potential biases due to a particular method of alignment, and any effect of potential alignment error, We generated a dataset to represent each sequence composed of antigenic site residues paired with a set of randomly selected residues for the HA1 region of each HA. These amino acids were extracted from each sequence, then the datasets containing the extracted residues representing each sequence were re-organized such that each dataset (representing a single sequence), now had new “nearest neighbors” in the data table. Values for Δ*abs,* ΔΔ*ASAtot,* Δ*ASAnp,* ΔΔASA_*pol*_, and Δ*Q* were recalculated for each amino acid in the dataset based on the new arrangement of sequences. The epitope residues for each HA were paired with datasets of randomly chosen residues. This resorting process was carried out twenty times to achieve a partially randomized arrangement of datasets. Statistical comparisons between the parameter values for the antigenic site amino acids and the randomly selected residues were performed both for the original alignments and the resorted datasets using Kruskal-Wallis ANOVA with Dunn’s post- test.

## Results and discussion

### Sequence alignment and parameterization

Amino acid sequences were aligned using the multiple protein sequence alignment tool MUSCLE. Since we wish to test the hypothesis that antibody selection is a key player in virus evolution, and this acts at the protein level, we elected to align amino acid rather than nucleic acid sequences, An alignment algorithm based on pairwise sequence comparison was chosen over other approaches because we wished to compare sequences on the basis of pairwise differences in values reflecting amino acid properties, and we reasoned that sequences aligned in such a fashion to minimize pairwise differences, as is the case with MUSCLE, would provide the most conservative approach, although we cannot rule out the possibility that potentially important sequence differences might be obscured. Amino acids in the alignment tables were then parameterized based on one of four properties: side chain size (measured by solvent-accessible surface area), hydrophobicity (measured by solvent-accessible non-polar surface area), hydrophilicity (measured by solvent-accessible polar surface area), or side-chain charge. Values pertaining to each property of interest were then compared mathematically to determine whether there was any trend in changes at a particular site in the protein (see Methods).

### Prediction of novel sites in potential antigenic sites in H1 and B HA

**Figure 1:**
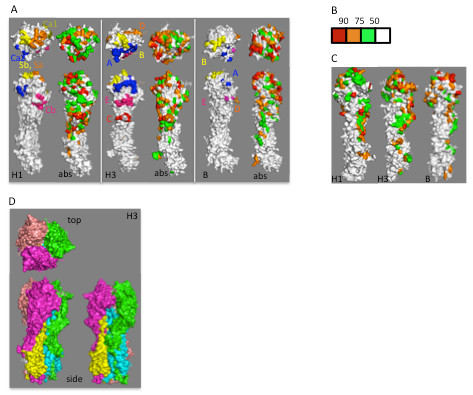
**Influenza HA antigenic sites.** (**a**) Comparison of previously described antigenic sites in influenza H1 H3, or B HA. For each HA, the structure to the left shows the previously defined antigenic site residues mapped onto a monomer an appropriate crystal structure, while the structure on the right shows residues colored according to the frequency of absolute change (i.e. any amino acid substituted with any other) in comparison with the same residue in the most closely related sequence (abs, see Materials and Methods, color code shown in panel b), viewed from the top (T) or side (S). The H1 structure shows antigenic residues [23] mapped onto the 3D structure of A/Puerto Rico/8/24 HA (PR8, PDB ID: 1RU7). Color scheme for antigenic sites: *Ca1*, olive; *Ca2*, blue; *Sb*, yellow; *Sa*, orange; *Cb*, red, as indicated by labels on the structure. Neutralizing antigenic sites [7] of influenza A H3 HA monomer mapped onto the crystal structure of A/X-31 HA (PBD ID: 2VIU). Color scheme: antigenic site *A*, blue; *B*, yellow; *C*, red; *D*, orange; *E*, magenta Antigenic sites in influenza B HA [24] mapped onto the 3D structure of B/Lee/40 HA (PDB ID: 1RFT), viewed from the top (T) or side (S). Color scheme: antigenic site *A*, blue; *B*, yellow; *c*, red; *D*, orange; *E*, magenta; base of receptor binding pocket, purple. (**b**) Color scheme indicating frequency of change: frequency below 50th percentile of all residues in HA1, white; between 50th and 75th percentile, green; between 75th and 90th percentile, orange; 90th percentile and above, red. (**c**). Views of H1, H3, and influenza B HA monomers from behind. (**d**) Crystal structure of H3 HA trimer (PBD ID: 2VIU), viewed from the top, the side, and along the intratrimer axis, shown for orientation.

Neutralizing antigenic sites have been described for human H1 [23,25], H3 [7,26], and influenza B [24] HA. For each HA, we calculated the average number of changes between neighboring aligned sequences (*Δabs*), and mapped these on to the surfaces of HA structures (Figure 1). There is reasonably good agreement between the previously described antigenic sites and residues with high *Δabs* values, especially those in the top 25^th^ percentile range (red and orange residues in Figure 1a). This is particularly true for H3, the human influenza HA best characterized at the antigenic level. Residues in each of the previously described H3 HA antigenic sites (*A-E*) are represented in the residues with the highest *Δabs* values (Figure 1a, Table 2), suggesting that pairwise sequence analysis for determining frequencies of change is a useful method for predicting residues that may be evolving in response to antibody selection. Somewhat unexpectedly, we also find rapidly changing residues on the rear face of the monomer, which would not be expected to be accessible to antibody, at least in the neutral pH conformation. Two of these residues, amino acids 220 and 229, have been shown to be under positive selection at the genetic level based on comparing rates of synonymous and non-synonymous nucleotide substitutions [17].

**Table 2:** Amino acids assigned to antigenic sites

**H1**^**a**^					
***Ca2***^***b***^	***Sb***	***Sa***	***H1C***^***c***^	***Ca1***	***Cb***
*132b*^*d,e*^, *133*, (140), (143), *144*, (145), *149,* (224), 225	156^f^, (159)^g^, *189*–*90*, 192–3, (196), *197,* (198)	(128), 129, *163*, 165, (166–7), *247-8*	*86*, *272*–*7*, *279*–*82*, *286*	(169), 173, (207), *212*, 240,*241*, 242, *243-5*	*51*, (74–5), 77, (78–9), (117), *149*, *255*–*6, 259-66*
**H3**^**h**^					
***A***	***B***		***C***	***D***	***E***
*121*, 122, (123), 124, (125), 126, (127), (129), *131*, (132), 133, (134), 135, (136), 137–8, *140*, 142, (143), 144–5, (146),	*155*, 156, (157), 158–60, 186, *188*–*9*, (190), *192*, 193, (194), (196), 197, *198*–*9*, *246-7*		*49*, 50, 53–4, *271*, *273*, 275, *276*, 278	*167*, 201–2, (203–6), *207*, *214*, *216*, (217– 8), 219–20, *222*–*3*, *225*–*7*, *242*	62, (63), *75*, *78*, (79–82), 83, *91*–*2*, *94*
**B**^**i**^					
***BA***	***BB1***^***j***^	***BB2***^***9***^	***BC***^***3***^	***BD***	***BE***
*136-7*, *141*, *146*, (147), *148*–*9*, 150, 154	*194*, 195, *196*, 197, 199, 200, *205-6*	*162*,*162a-c,*163, (164), *165*	*47-8*, *80*–*1*, *116*, *276*, *281*	*121-2*, 125, *126*–*7*, *129*, 179–80, *181*, *248*–*9*, *252-5*	*56*, 58, *68*–*9*, *71*, *73*, 75, *76*

^a^H1 numbered according to the amino acid position in A/Puerto Rico/8/34/Mount Sinai.

^b^Residues showing rates of change in the top 25% of all residues were assigned to previously described epitopes [7,23,24].

^c^Not defined in prior studies.

^d^Amino acid 132a deleted in A/Puerto Rico/8/34/Mount Sinai, but present in other strains prior to 1997.

^e^Residues assigned to this epitope in this work only are *italicized.*

^f^Residues assigned to this epitope in this work and in previous studies are underlined.

^g^Residues assigned to this epitope in previous studies not meeting our inclusion criteria are enclosed in parentheses.

^h^H3 HA residues numbered as for mature HA1 of A/Aichi/2/1968.

^i^Influenza B HA residues numbered as for B/Lee/40.

^j^Previous studies define a single epitope at the top of influenza B HA [24], but we have elected to divide this into two based on apparent functional differences based on the pattern of biophysical changes we observe.

When *Δabs* values were mapped onto the surface of the influenza H1 HA monomer from crystal structure and compared to antigenic sites described for A/Puerto Rico/8/34 (H1N1, Figure 1a), there is good agreement between residues showing high Δ*abs* values and the previously identified *Sb* and *Sa* antigenic sites on the top of the HA molecule ([23,25], yellow and orange, respectively), roughly akin to the *B* antigenic site of H3 HA. The *Ca2* antigenic site, below the receptor binding site (RBS), structurally analogous to the *A* site in H3 (blue in Figure 1a), shows some overlap with residues in this region showing high Δ*abs* values, but higher values are seen for neighboring residues that form part of a prominent projection immediately below the RBS. Overlap with the remaining previously-described H1 antigenic sites, *Ca1* and *Cb* (olive and red in Figure 1a) is less extensive. Additionally, high Δ*abs* values predict an additional antigenic site composed of shelf-like projection below the *Cb* antigenic site, analogous to the *C* antigenic site in H3. For ease of further discussion, we will refer to this as *H1C*. We note that a somewhat similar antigenic site in H1 HA has been reported elsewhere [27]. Residues assigned to each antigenic site are listed in Table 2. As for H3, H1 residues at the rear of the monomer are also changing relatively rapidly, and one of these, amino acid 98, has shown to be positively selected [18]. Strikingly, differences in Δ*abs* values between the *Sa* antigenic site on the top of the H1 monomer and non-antigenic site residues are not statistically significant, suggesting that the rate of change at this antigenic site is not high, even though the loss of a glycosylation site at this antigenic site seems to be a critical antigenic difference between “seasonal” H1N1 strains circulating between 1977 and 2008, and the pandemic “Swine-origin” 2009 H1N1 strains [28], possibly because this site might be constrained to preserve some unknown function. All other antigenic sites described are statistically significantly different from non-antigenic site residues in terms of Δ*abs* values.

When compared to a previous antigenic map of B HA [24], residues with high *Δabs* values match well with the best defined antigenic site, analogous to the influenza A H3 *B* and H1 *Sb* antigenic sites, lying above the RBS. Antigenic sites analogous to the H3 *BD*, and *E* antigenic sites were previously defined, some by as few as three residues. Based on high *Δabs*, our studies support the existence of important antigenic determinants in these areas of the molecule, and suggest the existence of two additional antigenic sites on influenza BHA. One is found on a shelf-like structure below the previously-described *E* antigenic site, analogous to the H3 *C* and *H1C* sites. For ease of discussion, we will refer to this as the *BC* antigenic site. On the top of the molecule, in addition to the previously described H3 *B*-like antigenic site, adjacent to this we observe a putative novel antigenic site analogous to the *Sa* site in H1. For ease of further discussion, we will refer to these as *BB1* and *BB2* antigenic sites, respectively. The *BB1* antigenic site consists of a “knob” of residues above the RBS, while *BB2* consists mainly of a ridge of rapidly changing residues across the top of the molecule.

### Site-specific differences in biophysical properties in H1 HA

**Figure 2:**
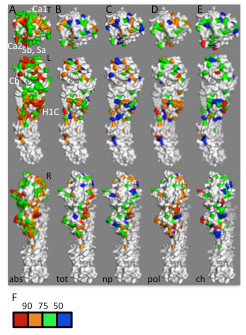
**Biophysical characteristics of influenza H1 HA antigenic sites.** (**a**) Rates of change at individual residues in H1 HA are shown from the top (T) and viewed along the left (L) and right (R) sides of the monomer. Note that structures marked T and L in panel a are identical to the structures marked “abs” in the leftmost section of Figure 1a with approximate positions of antigenic sites *Ca1*, *Ca2*, *Cb*, *Sa*, *Sb*, and *H1C* (see Table 2) indicated with white labels. Color-coding of surface residues is as described in Figure 1b. The most rapidly changing residues in H1 HA (75^th^ percentile and above; red and orange in panel *A*) were color-coded according to the average pairwise change in NCI values for *∆∆ASA*_*tot*_ (*tot*, panel **b**), *∆∆ASA*_*np*_ (*np*, panel **c**), *∆∆ASA*_*pol*_ (*pol*, panel **d**), or *∆Q* (*ch*, panel **e**). (**f**) Color scheme for panels b-e: residues whose absolute rate of change is lower than the 75^th^ percentile, white, residues in the top 25^th^ percentile in terms of absolute amino acid changes but whose change in the value of interest was below the 50th percentile of all residues in HA1, blue; values between 50th and 75th percentile, green; values between 50th and 90th percentile, orange; values above 90th percentile, red. Structure files used to generate panels *a*-*e*, viewable using PyMol, are available on line (Additional files 2, 3, 4, 5 and 6).

When biophysical properties of those residues in H1 undergoing most frequent changes (Δ*abs* values in the 75^th^ percentile and above) were examined, there are quite striking differences between different antigenic sites. Changes in NCI values for ΔΔ*ASA*_*tot*_ for the *Ca2SbSa*, and *H1C* antigenic sites were not statistically significant compared to changes in NCI values for ΔΔ*ASA*_*tot*_ for non-antigenic site residues in H1 HA (Figure 2, Table 3), suggesting that volume occupied by individual amino acids, and hence the shape of the surface in these regions associated with antibody binding, is relatively conserved. This suggests that the overall shape of these antigenic sites is not particularly important in antibody recognition, and so changes to the shape of the antigenic site do not confer a selective advantage. Alternatively, the shape of the antigenic site must be conserved to prevent loss of some other important function, such as binding of cell surface receptors or a putative co-receptors [29]. In contrast, two antigenic sites on the side of the trimer, *Ca1*, and *Cb,* did show statistically significant changes in ΔΔ*ASA*_*tot*_ NCI, suggesting that changes in the shape of the surface in this region is at least tolerated, if not advantageous due to disruption of antibody binding. The *Ca1* antigenic site is close to the trimer interface, so changes in shape might alter interactions between monomers, potentially affecting stability and influencing the pH of the transition to the fusion-active conformation. No significant changes in ΔΔ*ASA*_*np*_ NCI are found in any of the H1 HA antigenic sites. The *H1C* and *Cb* sites show significant differences in changes in NCI values for ∆∆*ASA*_*pol*_, and the *Ca2* antigenic site shows significant differences in ∆*Q* compared to non-antigenic site residues in HA1.

**Table 3:** Statistical comparison of antigenic sites to non-antigenic residues

**Antigenic Site^a^**	***p*^b^ antigenic site *vs.* non-antigenic site residues**				
	**Δ*abs***	**ΔΔ*ASAtot***	**ΔΔ*ASAnp***	**ΔΔ*ASApol***	**Δ*Q***
H1					
*Ca2*	*p* < 0.01	*NS*	*NS*	*NS*	*p* < 0.05
*Sb*	*p* < 0.001	*NS*	*NS*	*NS*	*NS*
*Sa*	*NS*	*NS*	*NS*	*NS*	*NS*
*H1C*	*p* < 0.001	*NS*	*NS*	*p* < 0.05	*NS*
*Ca1*	*p* < 0.05	*p* < 0.001	*NS*	*NS*	*NS*
*Cb*	*p* < 0.001	*p* < 0.05	*NS*	*p* < 0.01	*NS*
H3					
*A*	*p* < 0.001	*NS*	*NS*	*NS*	*NS*
*B*	*p* < 0.001	*NS*	*NS*	*NS*	*NS*
*C*	*p* < 0.001	*NS*	*NS*	*NS*	*NS*
*D*	*p* < 0.001	*NS*	*NS*	*NS*	*NS*
*E*	*p* < 0.01	*NS*	*NS*	*NS*	*NS*
B					
*BA*	*p* < 0.001	*p* < 0.001	*p* < 0.001	*p* < 0.001	*p* < 0.01
*BB1*	*p* < 0.001	*p* < 0.01	*p* < 0.001	*p* < 0.01	*p* < 0.001
*BB2*	*p* < 0.001	*p* < 0.01	*p* < 0.001	*p* < 0.01	*p* < 0.01
*BC*	*p* < 0.001	*p* < 0.01	*p* < 0.01	*p* < 0.01	*p* < 0.05
*BD*	*p* < 0.001	*p* < 0.001	*p* < 0.001	*p* < 0.001	*p* < 0.01
*BE*	*p* < 0.001	*p* < 0.05	*p* < 0.01	*NS*	*p* < 0.05

^a^See Table 2

^b^Statistics: Kruskal Wallis one-way ANOVA (non-parametric) with Dunn’s post-test

^c^Non-antigenic site residues are all residues in HA1 not assigned to a particular antigenic site

**Figure 3:**
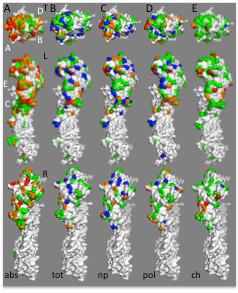
**Biophysical characteristics of influenza H3 HA antigenic sites**. (**a**) Rates of change at individual residues in H3 HA are shown from the top (T) and viewed along the left (L) and right (R) sides of the monomer. Note that structures marked T and L in panel a are identical to the structures marked “abs” in the middle section of Figure 1A, with approximate positions of antigenic sites *A*, *B*, *C*, *D*, and *E* indicated with white labels. Color-coding of surface residues is as described in Figure 1b. The most rapidly changing residues in H1 HA (75^th^ percentile and above; red and orange in panel **a**) were color-coded according to the average pairwise change in NCI values for *∆∆ASA*_*tot*_ (*tot*, panel **b**), *∆∆ASA*_*np*_ (*np*, panel **c**), *∆∆ASA*_*pol*_ (*pol*, panel **d**), or *∆Q* (*ch*, panel **e**). Color scheme for panels *b*-*e* as in Figure 2f. Structure files used to generate panels *a*-*e*, viewable using PyMol, are available on line (Additional files 7, 8, 9, 10 and 11).

**Figure 4:**
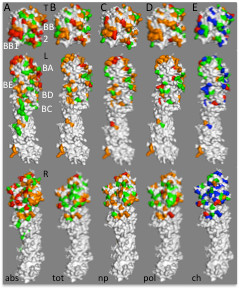
**Biophysical characteristics of influenza B HA antigenic sites.** (**a**) Rates of change at individual residues in influenza B HA are shown from the top (T) and viewed along the left (L) and right (R) sides of the monomer. Note that structures marked T and L in panel a are identical to the structures marked “abs” in the rightmost section of Figure 1a, Approximate positions of antigenic sites BA, BB1, BB2, BC, BC, BD, and BE (see Table 2) are indicated with white labels. Color-coding of surface residues is as described in Figure 1b. The most rapidly changing residues in H1 HA (75^th^ percentile and above; red and orange in panel *a*) were color-coded according to the average pairwise change in NCI values for *∆∆ASA*_*tot*_ (*tot*, panel **b**), *∆∆ASA*_*np*_ (*np*, panel **c**), *∆∆ASA*_*pol*_ (*pol*, panel **d**), or *∆Q* (*ch*, panel **e**). Color scheme for panels *b*-*e* as in Figure 2f. Structure files used to generate panels *a*-*e*, viewable using PyMol, are available on line (Additional files 12, 13, 14, 15 and 16).

### Biophysical properties of frequently changing H3 residues

Sequences of HA genes from 958 human H3N2 influenza isolates were analysed as described above (Figure 3, Table 2,3). Unlike H1, we did not observe statistically significant differences in ∆∆*ASA*_*tot*_, ∆∆*ASA*_*np*_, or ∆∆*ASA*_*pol*_ NCI between any of the H3 antigenic sites and non-antigenic site residues. Although the potential importance of charge in evolution of H3 antigenic sites has also been recently suggested [30], we did not find statistically significant differences in ∆*Q* between rapidly changing residues and non-antigenic site residues for any H3 antigenic site. Strikingly, some of the least conservative changes occur in residues within antigenic site *D* and at the rear of the trimer (Figure 1d), in areas of the molecule at least partially occluded by the neighboring monomer in the 3D structure. It has been suggested that these changes affect antibody binding at a distance by changing the conformation at the surface [26]. Other studies demonstrate that the trimer may adopt a more open conformation than seen in the crystal structures at least transiently, exposing these residues to antibody [31]. Thus, changes in the region of the trimer interface may act to increase or decrease the stability of the trimer, and covariation of residues interacting in the interface between neighboring monomers might be expected to occur. Alternatively, the rate of change of residues expected to be occluded based on the crystal structure may represent a background rate of amino acid change, and that all areas of the molecule undergoing change at lower rates are actually undergoing negative selection to maintain important functions such as interaction with alternate receptors or putative co-receptors [29,32].

### Differences in biophysical properties define separate adjacent antigenic sites in B HA

HA genes from 209 influenza B isolates were also studied (Figure 4, Tables 2-3). Unlike influenza A H1 and H3 HAs, NCI values for ΔΔ*ASA*_*tot*_*,* ΔΔ*ASA*_*np*_, ΔΔ*ASA*_*pol*_, and ∆*Q* are significantly different between each of the antigenic sites residues and non-antigenic site residues for all but ΔΔ*ASA*_*pol*_ NCI values at antigenic site *BE*. These findings suggest that changes in BHA antigenic sites may be more likely to confer selective advantage than those occurring in H1 and H3 HAs.

### Observed changes in biophysical properties are dependent on alignment

To determine whether our findings were dependent upon the quality of the sequence alignment, NCI values for antigenic site residues were compared to a randomly chosen set of ten residues from the same HA (Table 4). The tables of sequences, with each sequence now represented by a dataset comprising the antigenic site residues (Table 2) and the randomly chosen residues, were then rearranged such that each sequence dataset now had new sequences as nearest neighbors, compared to its position in the original alignment. NCI values for Δ*abs*, ΔΔ*ASA*_*tot*_*,* ΔΔ*ASA*_*np*_, ΔΔ*ASA*_*pol*_, and ∆*Q* were calculated for each amino acid position before rearrangement, and after twenty rounds of resorting, which we believe represents a partial randomization of the sequence order. In many cases, the degree of statistical significance differed between the same datasets in the original alignment and following partial randomization (Table 4). The fact that the statistical significance is altered when the data obtained reflect an alignment where the nearest neighbor sequences are not necessarily the most closely related suggests both that our analysis is yielding important information about changes between the most closely related sequences, and that our conclusions might be skewed if the alignment of sequences is poor.

**Table 4:** Effect of partial randomization of sequence dataset on antigenic site statistics

**Antigenic Site^a^**	***p*^b^ from aligned sequences^c^ (*p*^*b*^ from resorted sequences^d^)**				
	**Δ*abs***	**ΔΔ*ASAtot***	**ΔΔ*ASAnp***	**ΔΔ*ASApol***	**Δ*Q***
H1 epitope residues *vs.* random^e^					
*Ca2*	*p* < 0.05 (*NS*)	*NS* (*NS)*	*NS* (*NS)*	*NS* (*NS)*	*p* < 0.05 (*p* < 0.05)
*Sb*	*p* < 0.05 (*p* < 0.01)	*NS* (*NS)*	*NS* (*NS*)	*NS* (*NS)*	*p* < 0.05 (*NS*)
*Sa*	*NS* (*p* < 0.05)	*NS* (*NS)*	*NS* (*NS)*	*NS* (*NS)*	*NS* (*NS*)
*H1C*	*p* < 0.01 (*p* < 0.05)	*NS* (*NS)*	*NS* (*NS)*	*NS* (*NS)*	*NS* (*NS*)
*Ca1*	*NS* (*NS)*	*NS* (*p* < 0.01)	*NS* (*NS)*	*NS* (*NS)*	*NS* (*NS*)
*Cb*	*NS* (*NS)*	*NS* (*NS)*	*NS* (*NS)*	*NS* (*NS)*	*NS* (*NS*)
H3 epitope residues *vs.* random^f^					
*A*	*NS* (*p* < 0.05)	*NS* (*NS*)	*NS* (*NS*)	*NS* (*NS*)	*NS* (*NS*)
*B*	*p* < 0.05 (*p* < 0.01)	*NS* (*NS*)	*NS* (*NS*)	*NS* (*NS*)	*NS* (*NS*)
*C*	*NS* (*NS*)	*NS* (*NS*)	*NS* (*NS*)	*NS* (*NS*)	*NS* (*NS*)
*D*	*p* < 0.05 (*NS*)	*NS* (*NS*)	*NS* (*NS*)	*NS* (*NS*)	*NS* (*NS*)
*E*	*NS* (*NS*)	*NS* (*NS*)	*NS* (*NS*)	*NS* (*NS*)	*p* < 0.05 (*NS)*
B HA epitope residues *vs.* random^g^					
*BA*	*NS* (*p* < 0.01)	*NS* (*NS)*	*NS* (*NS)*	*NS* (*NS)*	*p* < 0.05 (*NS*)
*BB1*	*NS* (*p* < 0.01)	*NS* (*NS)*	*NS* (*p* < 0.05)	*NS* (*NS)*	*p* < 0.001 (*p* < 0.05)
*BB2*	*NS* (*p* < 0.05)	*NS* (*NS)*	*NS* (*NS)*	*NS* (*NS)*	*p* < 0.05 (*NS*)
*BC*	*NS* (*NS)*	*NS* (*NS)*	*NS* (*NS)*	*NS* (*NS)*	*p* < 0.05 (*NS*)
*BD*	*NS* (*NS)*	*NS* (*NS)*	*NS* (*NS)*	*NS* (*NS)*	*p* < 0.05 (*NS*)
*BE*	*NS* (*NS)*	*NS* (*NS)*	*NS* (*NS)*	*NS* (*NS)*	*NS* (*NS*)

^a^See Table 2

^b^Probability determined from comparison of antigenic site residues to randomly selected residues (see below) determined using Kruskal-Wallis ANOVA

^c^Amino acid sequences aligned using MUSCLE. See Additional file 1 for resultant sequence alignment.

^d^Antigenic site residues, along with a set of randomly selected residues (below), were extracted from each sequence, then the datasets containing the extracted residues representing each sequence were re-organized and Δ*abs,* ΔΔ*ASAtot,* ΔΔ*ASAnp, and* Δ*Q* recalculated for each amino acid in the dataset based on the new arrangement of sequences (see Methods)

^e^Randomly selected H1 residues: 2, 19, 23, 76, 84, 94, 117, 121, 178, 223, 301, 326

^f^Randomly selected H3 residues: 12, 72, 111, 123, 139, 179, 222, 289, 312, 328

^g^Randomly selected influenza B HA residues: 4, 17, 36, 47, 78, 152, 196, 222, 251, 273, 300, 304

### Comparison of changes in biophysical properties to other techniques to identify evolutionarily important residues

**Figure 5:**
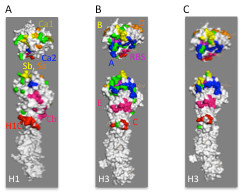
**Comparison of antigenic site residues with residues under positive selection.** (**a**) Comparison of H1 antigenic site residues described in this study (Table 2, color-coded as for Figure 1a) with residues positively selected in human H1N1 viruses [18] colored lime green. (**b**) Comparison of H3 antigenic site residues described in this study (Table 2, color-coded as for Figure 1a except that the H1C epitope defined in this study is shown in red) with residues predictive of novel lineages in human H3N2 viruses [33] colored lime green. To orient the reader, the receptor binding site (RBS) has been labeled in purple. (**c**) Comparison of H3 antigenic site residues described in this study (Table 2, color-coded as for Figure 1a) with directionally selected residues in human H3N2 viruses [34] colored lime green. Structure files showing epitopes, viewable using PyMol, are available on line (Additional files 17 and 18).

We wished to compare our results to those of others who have attempted to identify residues in influenza HA which might have evolutionarily predictive value (Table 5, Figure 5). A recent study of human seasonal H1N1 viruses identified eight residues in HA1 which were apparently under positive selection [18]. Of these, all but one residue is also found in our dataset of amino acid residues (Table 2), and statistically significant differences are found between this dataset and the non-antigenic site residues from H1 HA1. Amino acid 98, the lone residue not assigned to an antigenic site in our studies is highly variable, but found on the solvent-exposed surface on the rear of the monomer. Studies of residues which were changed in viruses forming new branches within the H3N2 HA phylogenetic tree identified a group of 19 residues which seemed to be predictive of forming a new branch [33]; of these, all but three are also assigned to antigenic sites in our study. Two of these (190 and 194) are adjacent to the receptor binding site and do not change at sufficiently high frequency to meet our inclusion criteria, and the remaining residue (262) is solvent exposed on the lip of the monomer at the trimer interface. This dataset is statistically significantly different from the H3 HA1 non-antigenic site residues in terms of the absolute frequency of amino acid change, but not in any other quantity examined. We also compared our data to a dataset of sites in H3 HA1 undergoing directional selection, another means of identifying accelerated substitutions at a specific site [34]. As for the residues identified in [18] and [33], many of the residues identified by this technique are also identified as antigenic site residues in our analysis. Unlike the residues identified by Bush *et al.*[33] and antigenic site residues from our methodology, we observe statistically significant differences between the dataset of directionally selected residues [34] and non-antigenic site residues for both ∆*abs* and NCI values for ∆*Q.* We note that a large number of amino acids are invariant in our dataset, particularly in H1 and influenza B. For those residues making critical structural interactions, this is presumably the result of negative selection to maintain structural integrity, but for those residues on the surface it is difficult to distinguish between the effects of negative selection to maintain a previously unappreciated function and the background rate of mutation in the absence of positive selection.

**Table 5:** Changes in biophysical properties of positively-selected or “predictive” amino acids

	**aa**	**Δ*abs***	**ΔΔ*ASAtot***	**ΔΔ*ASAnp***	**ΔΔ*ASApol***	**Δ*Q***
Positively Selected in H1*.*^a,b^	**86**^**c**^**,** 98^d^**, 144, 163, 165, 189, 190, 225**	*p* < 0.001^e^	*NS*	*p* < 0.05	*NS*	*p* < 0.001
“Predictive” in H3^f^	**121, 124, 133, 135, 138, 142, 145, 156, 158, 186,** 190^g^**, 193,** 194^g^**, 197, 201, 226,** 262^h^**, 275**	*p* < 0.001	*NS*	*NS*	*NS*	*NS*
Directionally selected in H3.^i,b^	45, **135, 145, 155, 158,** 229, 248	*p* < 0.001	*NS*	*NS*	*NS*	*p* < 0.01

^a^See [17]

^b^Amino acid numbers converted to conform to system described in Table 2

^c^Bolded numbers indicate that amino acid is defined as an epitope residue in our analysis (see Table 2)

^d^Solvent exposed at the rear of the H1 monomer

^e^Statistical comparison to non-epitope residues from HA1 (Kruskal Wallis one-way ANOVA (non-parametric) with Dunn’s post-test)

^f^See [33]

^g^Receptor binding site residue

^h^Solvent exposed on edge of monomer

^i^See [34] Residues not in HA1 are excluded from this analysis

### Role of alteration in biophysical properties in antibody-mediated selection of variant viruses

Insights into the mechanism of antibody binding have been derived from structural, biophysical, and biochemical characterization of antibody-antigen pairs [14], particularly for hen-egg lysozyme and anti-idiotypic antibodies (reviewed in [35]), and influenza A HA (reviewed in [36]) and NA [10,13,37,38]. Changes in shape of the antigenic sites due changes in the volumes of individual side-chains were monitored by examining *ΔΔASA*_*tot*_. Larger NCI values for *ΔΔASA*_*tot*_ suggest that an amino acid with a small side-chain surface area has been replaced with a larger amino acid or *vice-versa*. The biophysical quantities *ΔΔASA*_*np*_*, ΔΔASA*_*pol*_*,* and *ΔQ* measure the propensity of residues to participate in certain kinds of interactions. Charged residues will interact with residues of opposite charge and be repelled by residues of like charge. Charged and polar residues can also participate in hydrogen bonding, either with water molecules or with other proteins. Hydrophobic interactions between non-polar surfaces are important in protein-protein interactions by contributing to positive entropy to favor the energetics of the bound state [22] and hydrophobic surfaces are a feature of at least some antibodies showing evidence of affinity maturation [35]. However, solvent-exposed hydrophobic surfaces are energetically unfavorable.

### Changes in shape may drive evolution of some antigenic sites

Statistically significant changes in *ΔΔASA*_*tot*_ NCI values were seen for antigenic sites on the side of H1 HA (*Cb* and *Ca1*) and for all antigenic sites described for influenza B HA, but not for antigenic sites at the top of the H1 HA (*Sb* and *Sa*) or at the trimer interface (*Cb*), or for any antigenic site in H3 HA. Thus, the *ΔΔASA*_*tot*_ NCI values we measured suggest that the shape of the surface in the antigenic sites is altered significantly by the accumulation of mutations for some antigenic sites, and thus changes in the overall shape of these antigenic site may contribute to escape from antibody binding. For those antigenic sites not showing significant differences in *ΔΔASA*_*tot*_*,* such as the *Sb* and *Sa* antigenic sites of on the top of H1, the shape of the surface may be critical to maintaining other hitherto unappreciated functions in virus binding or entry.

### Changes in thermodynamic properties may influence antibody escape

Statistically significant changes in *ΔΔASA*_*np*_ NCI values were found for all antigenic sites in influenza B HA, and for previously-described positively selected residues in H1 HA. The fact that the influenza B HA antigenic sites have some hydrophobic character might indicate that they play some other role in the function of HA, so there may be important functional reasons for hydrophobic residues to be retained. Antibody binding sites studied to date at the structural and biophysical level seem to fall into at least two classes, the first, where the antigenic site consists of a central core area of hydrophobic residues, often surrounded by an outer ring of hydrophilic amino acids, and a second where hydrophilic residues and immobilized water molecules seem to play an important role. In the first situation, so called “O-ring” epitopes, much of the binding energy is contributed by the increase in entropy due to the liberation of the highly ordered water molecules at the hydrophobic residues in both antibody and antigen. Thus, mutation of hydrophobic residues in the antigenic site would be expected to reduce the binding energy of the antibody-antigen complex, as has be shown *in vitro*[11,39]. We note that many of the positively selected residues identified by Li *et al.,* which as a group show significant differences in *ΔΔASA*_*np*_ NCI values compared to non-antigenic site residues in H1 HA (Table 5), are also identified in our study. These residues are mainly found in the *Ca2Sb*, and *Sa* antigenic sites. In the *Sb*, and *Sa* antigenic sites, positively selected residues are clustered together towards near center of our antigenic sites, suggesting that these amino acids may act as the hydrophobic core of “O-ring” like epitopes (Figure 2).

Biophysical and structural studies show that charge-charge interactions (“salt bridges”) can make critical contributions to both the extent and rate of antibody binding [40], thus it is logical that changes in charge within an antigenic site may confer a selective advantage, as seen in the H1 *Ca2* and influenza B HA antigenic sites. The loss of a critical charged residue would be expected to have a deleterious effect on both rate and extent of antibody binding, and the gain of a novel charged residue could either prevent antibody binding due to electrostatic repulsion or alter the rate of binding by altering “electrostatic steering” required for correct alignment of an antibody with its cognate antigenic site (see [41] for review).

### Forces shaping evolution of influenza HA may vary between subtypes and antigenic sites

Differences between the different HAs, and between antigenic sites of the same HA molecule may suggest that the “rules” for selecting changes at these sites may be different. The rates of change of amino acid identity were significant for all H3 and influenza B HA antigenic sites compared to non-antigenic site residues, and for all but the *Sa* antigenic site of H1 HA. This result is somewhat surprising, given that an important structural difference in this antigenic site between the pandemic “Swine-origin” H1N1 influenza virus emerging in 2009 and prior seasonal H1N1 apparently played an important role the susceptibility of the many people born after 1957 to the pandemic virus [28]. No statistically significant changes in the other quantities studied were observed for H3 HA, suggesting that the antibody repertoire against H3 HA, if responsible for selecting the changes observed, is sufficiently discriminatory that even highly conservative amino acid substitutions are sufficient to confer a selective advantage. Interestingly, some residues on the surface of HA monomer apparently undergoing rapid change may not be antibody accessible, at least based on the available crystal structures, suggesting their evolution may be controlled by other factors. This is particularly true of the rapidly changing residues on the “rear” of the HA monomer, which would not be expected to be solvent exposed in the neutral pH trimer form, although there is good evidence to suggest that the HA trimer is less rigid *in vivo* than expected from available crystallographic and electron microscopy data, allowing the trimer structure to open and close [31]. These residues may vary simply because they are not under negative selection since they would not be expected to be required to participate in any of the known functions of HA and are not involved in stabilizing its secondary, tertiary, or quaternary structure.

### Possible implications for influenza evolution and immunity

Our data raise several important issues in understanding the function of influenza HA and the host immune system. First, there appear to be important differences between evolution of H3 HA and that of H1 and influenza B HA. This suggests immune responses to H3 HA may be functionally different from the immune responses to H1 and influenza B. Differences in the role of antibody selection between influenza B and H3N2 viruses have been proposed previously [42]. Our data suggest that even conservative structural or biophysical changes in H3 HA antigenic sites may be sufficient to confer a selective advantage. Influenza B and H1 HAs may also be more subject to structural or functional constraints, so fewer kinds of changes are permitted. A second possibility is that escape from antibody neutralization may not be a significant positive selection for H3N2 viruses *in vivo*, and changes in the neutralizing antigenic sites may be selected because they act in concert with other changes in replication in order to generate more fit progeny, as observed with recent human H3N2 isolates [43].

The specific kinds of changes observed in antigenic sites in influenza B and H1 HAs, may also suggest that the antibody repertoires specific for these sites is more restricted than for H3N2 viruses, and thus a particular type of change may be reflected in the antibody response of many individuals. The primary anti-influenza antibody response in humans may not be truly polyclonal, at least against influenza B and H1 HAs. Instead, certain heavy and/or light chain rearrangements and combinations may be more likely to confer tight binding to individual antigenic sites. Studies in humans vaccinated against H1N1 and H3N2 also showed that the primary response is highly restricted, with some donors having only small numbers of unique V_H_ and V_L_ rearrangements represented but showing evidence of significant diversification due to somatic hypermutation [44]. Similarly, studies in BALB/c mice immunized with influenza A/Puerto Rico/8/34 (PR8) showed that certain heavy and light chain genes, and particular V_H_-V_L_ combinations were overrepresented in the primary antibody response [45-47], with more than 50% of the antibodies in the primary response targetted to a particular antigenic site sharing a single V_L_ gene [46]. Interestingly, those antibodies most abundant in the primary response were not as frequent in the secondary response, which showed a broader representation of V_H_ and V_L_ genes. Thus, the apparent differences in behavior we observe at different antigenic sites could represent the effects of positive selection by a set of primordial anti-influenza antibodies overrepresented in the primary antibody response.

If positive selection by antibody does indeed play an important role, understanding how influenza virus persists in a large and outbred population with a highly diverse immune system, such as humans, presents something of a conundrum. The viruses circulating each year are closely related both to each other and to the viruses circulating in the previous year. It has been suggested that certain individuals in the population play a disproportionate role in the spread of influenza [48]; such “superspreaders”, should they exist, might also play a role as “superselectors” in modulating the virus repertoire in the human population. The existence of some sort of primordial antibody response where a particular V_H_, V_L_, or V(D)J rearrangement predominates would also explain apparent differences in behavior between different antigenic sites in the same molecule, since each antigenic site would be under the selection of a different set of primordial antibodies that are consistent from individual to individual. Thus, influenza viruses evolving to escape this primordial response in one individual would now have a selective advantage in other human hosts.

The role of antibody selection remains a critical open question in understanding evolution of influenza virus in the human population. Our data suggest that the relative contribution of positive selection for antibody escape may vary from subtype to subtype and site to site. Other data suggest that there is a complex interplay between antigenicity and receptor utilization. For example, studies comparing infection of immunized mice with mouse-adapted influenza virus gave rise to numerous HA mutations which simultaneously altered both receptor binding and antibody neutralization [49]. Analyses of clinical H3N2 viruses from 2003 to 2008 indicated that these viruses had become progressively restricted in terms of the types of sialic acids bound, correlating with a decreased requirement for receptor-matched NA activity [50-52]. Since, as seen in HA, antigenic sites on NA are also located on the lip of the receptor binding pocket [8], adjustments in receptor binding could either drive or result from changes in antigenicity of HA, or even changes in NA. Finally, in the context of the polyclonal antibody response, the role of alterations in virus replication or innate immunity cannot be discounted [53].

## Conclusions

We have attempted to integrate an understanding of the role of protein structure and the thermodynamics of protein-protein interactions into evolutionary studies of influenza virus. Our studies indicate important and surprising differences in the evolution of different influenza HAs, and different antigenic sites within these molecules in humans, possibly due to differences in the immune response mounted to these viruses. Some antigenic sites show evidence that changes affecting specific biophysical properties may play critical roles in selecting novel influenza variants. Our findings may allow development of models to predict, or at least assess the importance of novel influenza strains in the future, enhancing the effectiveness of vaccine design.

## Abbreviations

∆ASA_np_: Side-chain non-polar surface area; ∆∆ASA_np_: change in side-chain non-polar surface area; ∆ASA_pol_: side-chain polar surface area; ∆∆ASA_pol_: change in side-chain polar surface area; ∆ASA_tot_: total solvent-exposed surface area due to the side-chain; ∆∆ASA_tot_: change in total solvent-exposed surface area due to the side-chain; HA: hemagglutinin; H1: hemagglutinin subtype 1; H3: hemagglutinin subtype 3; NCBI: National Center for Biotechnology Information; NA, neuraminidase; N1: neuraminidase subtype 1; N2, neuraminidase subtype 2; NCI: normalized change index; Q, side-chain net charge at pH 7.0; ∆Q: Change in side-chain net charge at pH 7.0; RBS: Receptor binding site.

## Competing interests

The authors declare that they have no competing interests.

## Authors’ Contributions

SJS conceived and designed the study, performed alignments, parameterized data, and database manipulations, interpreted the data, and drafted the manuscript. LBP performed alignments, parameterized data, and database manipulations. Both authors reviewed and approved the final version of the manuscript.

## Supplementary Material

Additional file 1 Multiple Sequence Alignments for influenza H1, H3, and B HA used in this study. Click here for file

Additional file 2 H1 HA structure color-coded to indicate ∆abs values. Click here for file

Additional file 3 H1 HA structure color-coded to indicate ∆∆ASA_tot_ values. Click here for file

Additional file 4 H1 HA structure color-coded to indicate ∆∆ASA_np_ values. Click here for file

Additional file 5 H1 HA structure color-coded to indicate ∆∆ASA_pol_ values. Click here for file

Additional file 6 H1 HA structure color-coded to indicate ∆Q values. Click here for file

Additional file 7 H3 HA structure color-coded to indicate ∆abs values. Click here for file

Additional file 8 H3 HA structure color-coded to indicate ∆∆ASA_tot_ values. Click here for file

Additional file 9 H3 HA structure color-coded to indicate ∆∆ASA_np_ values. Click here for file

Additional file 10 H3 HA structure color-coded to indicate ∆∆ASA_pol_ values. Click here for file

Additional file 11 H3 HA structure color-coded to indicate ∆Q values. Click here for file

Additional file 12 Influenza B HA structure color-coded to indicate ∆abs values. Click here for file

Additional file 13 Influenza B HA structure color-coded to indicate ∆∆ASA_tot_ values. Click here for file

Additional file 14 Influenza B HA structure color-coded to indicate ∆∆ASA_np_ values. Click here for file

Additional file 15 Influenza B HA structure color-coded to indicate ∆∆ASApol values. Click here for file

Additional file 16 Influenza B HA structure color-coded to indicate ∆Q values. Click here for file

Additional file 17 H1 HA structure color-coded to indicate epitopes and positively selected residues. Click here for file

Additional file 18 H3 HA structure color-coded to indicate epitopes and “predictive” residues. Click here for file
